# Endovascular treatment in anterior circulation stroke beyond 6.5 hours after onset or time last seen well: results from the MR CLEAN Registry

**DOI:** 10.1136/svn-2020-000803

**Published:** 2021-04-07

**Authors:** Luuk Dekker, Esmee Venema, F Anne V Pirson, Charles B L M Majoie, Bart J Emmer, Ivo G H Jansen, Maxim J H L Mulder, Robin Lemmens, Robert-Jan B Goldhoorn, Marieke J H Wermer, Jelis Boiten, Geert J Lycklama à Nijeholt, Yvo B W E M Roos, Adriaan C G M van Es, Hester F Lingsma, Diederik W J Dippel, Wim H van Zwam, Robert J van Oostenbrugge, Ido R van den Wijngaard, the MR CLEAN Registry investigators, Jan Albert Vos

**Affiliations:** 1 Neurology, Leiden University Medical Centre, Leiden, The Netherlands; 2 Neurology, Erasmus MC, Rotterdam, The Netherlands; 3 Public Health, Erasmus MC, Rotterdam, The Netherlands; 4 Neurology, Maastricht University Medical Centre+, Maastricht, The Netherlands; 5 Radiology and Nuclear Medicine, Amsterdam University Medical Centre, Location AMC, Amsterdam, The Netherlands; 6 Neurology, University Hospitals Leuven, Leuven, Belgium; 7 Neurosciences, Experimental Neurology and Leuven Brain Institute, University of Leuven, Leuven, Belgium; 8 VIB, Center for Brain & Disease Research, Laboratory of Neurobiology, Leuven, Belgium; 9 Neurology, Haaglanden Medical Centre, Den Haag, The Netherlands; 10 Radiology, Haaglanden Medical Centre, Den Haag, The Netherlands; 11 Neurology, Amsterdam University Medical Centre, Location AMC, Amsterdam, The Netherlands; 12 Radiology, Leiden University Medical Centre, Leiden, The Netherlands; 13 Radiology, Maastricht University Medical Centre+, Maastricht, The Netherlands

**Keywords:** Thrombectomy, Stroke

## Abstract

**Background:**

Randomised controlled trials with perfusion selection have shown benefit of endovascular treatment (EVT) for ischaemic stroke between 6 and 24 hours after symptom onset or time last seen well. However, outcomes after EVT in these late window patients without perfusion imaging are largely unknown. We assessed their characteristics and outcomes in routine clinical practice.

**Methods:**

The Multicenter Randomized Clinical Trial of Endovascular Treatment for Acute Ischemic Stroke in the Netherlands Registry, a prospective, multicentre study in the Netherlands, included patients with an anterior circulation occlusion who underwent EVT between 2014 and 2017. CT perfusion was no standard imaging modality. We used adjusted ordinal logistic regression analysis to compare patients treated within versus beyond 6.5 hours after propensity score matching on age, prestroke modified Rankin Scale (mRS), National Institutes of Health Stroke Scale, Alberta Stroke Programme Early CT Score (ASPECTS), collateral status, location of occlusion and treatment with intravenous thrombolysis. Outcomes included 3-month mRS score, functional independence (defined as mRS 0–2), and death.

**Results:**

Of 3264 patients who underwent EVT, 106 (3.2%) were treated beyond 6.5 hours (median 8.5, IQR 6.9–10.6), of whom 93 (87.7%) had unknown time of stroke onset. CT perfusion was not performed in 87/106 (80.2%) late window patients. Late window patients were younger (mean 67 vs 70 years, p<0.04) and had slightly lower ASPECTS (median 8 vs 9, p<0.01), but better collateral status (collateral score 2–3: 68.3% vs 57.7%, p=0.03). No differences were observed in proportions of functional independence (43.3% vs 40.5%, p=0.57) or death (24.0% vs 28.9%, p=0.28). After matching, outcomes remained similar (adjusted common OR for 1 point improvement in mRS 1.04, 95% CI 0.56 to 1.93).

**Conclusions:**

Without the use of CT perfusion selection criteria, EVT in the 6.5–24-hour time window was not associated with poorer outcome in selected patients with favourable clinical and CT/CT angiography characteristics. randomised controlled trials with lenient inclusion criteria are needed to identify more patients who can benefit from EVT in the late window.

## Introduction

Following the publication of the first positive randomised controlled trials in endovascular treatment (EVT) for acute ischaemic stroke,^1^ guidelines recommended EVT in patients with a large vessel occlusion of the anterior circulation if treatment could be initiated within 6 hours from symptom onset.^2 3^ However, this paradigm leads to exclusion from EVT of a significant number of patients who had a stroke, since an estimated 30% of them present between 6 and 24 hours after symptom onset or after the time that patients were last seen well (LSW).^4^ The 6-hour time window for EVT was based on the inclusion criteria used in previous trials and the observation that treatment effect decreased over time to non-significant at a time point beyond 6 hours.^5 6^ The HERMES collaborators demonstrated that treatment effect was still present up to 7.3 hours in included patients.^5^ However, some of the included trials used additional advanced imaging for patient selection. Therefore, these results cannot be generalised to all patients who had a stroke presenting beyond the 6-hour time window.

In 2018, two randomised controlled trials, DEFUSE 3 and DAWN, showed that EVT is safe and effective in patients treated in an extended time window of up to 16 or 24 hours after symptom onset or time LSW.^7 8^ In these trials, selection of patients was predominantly based on perfusion imaging. Following the publication of these trials, EVT for patients beyond 6 hours from symptom onset or LSW who meet the inclusion criteria of these trials with mandatory perfusion imaging is also recommended.^9^ However, on a global scale, software with automated perfusion analysis is currently offered to a minority of late window patients who had a stroke. Furthermore, the inclusion criteria used in these trials exclude a majority of late window patients of whom it is yet unknown if they may benefit from EVT.^4^ We describe characteristics, outcome and safety of patients treated with EVT within and beyond the 6.5-hour time window in clinical practice prior to the current guidelines with DAWN/DEFUSE 3 paradigms.

## Methods

### Patient inclusion

We analysed patients from the Multicenter Clinical Registry of Endovascular treatment for Acute ischaemic stroke in the Netherlands (MR CLEAN Registry), which was a national, prospective, observational stroke registry in 16 intervention centres that perform EVT in the Netherlands. Registration of patients treated with EVT started directly after the original Multicenter Randomized Clinical Trial of Endovascular Treatment for Acute Ischemic Stroke in the Netherlands (MR CLEAN) trial was finished, and was used to study safety and efficacy of EVT in routine clinical practice.^10^ All patients in whom EVT was considered indicated and who underwent an arterial groin puncture were included. EVT consisted of arterial catheterisation with a microcatheter to the level of the occlusion, followed by mechanical thrombectomy and/or thrombus aspiration, with or without delivery of a thrombolytic agent. The method of EVT was left to the discretion of the treating physician.

An independent core lab, blinded for all outcome measures, assessed imaging data, including early ischaemic changes with the Alberta Stroke Programme Early CT Score (ASPECTS) on baseline non-contrast CT,^11^ collateral status on baseline single phase CT angiography,^12^ and reperfusion status on digital subtraction angiography after EVT. Reperfusion was scored with the extended Thrombolysis in Cerebral Infarction (eTICI) score, which ranges from grade 0 (no reperfusion) to grade 3 (complete reperfusion).^13^ Data were collected before publication of the DAWN and DEFUSE 3 trials, and CT perfusion was not a standard imaging modality in our registry. None of the participating stroke centres used RAPID software in this time period. No records were kept concerning the use of CT perfusion imaging in patients who underwent EVT between March 2014 and June 2016, but this was documented for patients treated after June 2016 and for all late window patients. MR diffusion weighted imaging was performed only in two late window patients and therefore not used in the current analysis.

In the present study, we included all patients with an intracranial proximal occlusion in the anterior circulation (intracranial carotid artery, middle cerebral artery (M1/M2) or anterior cerebral artery (A1/A2)) demonstrated on CT angiography, treated with EVT between 16 March 2014 and 1 November 2017 in centres that had participated in the MR CLEAN trial. Exclusion criteria were age <18 years or an unknown time between onset or LSW and groin puncture. Although the recommended time window for EVT at the time of this study was 6 hours,^9^ in clinical practice the start of procedure (time of groin puncture) was sometimes slightly delayed due to logistical reasons. Therefore, late window patients were defined as patients in whom treatment was started at or beyond 6.5 hours after documented symptom onset or after time LSW in case of unknown stroke onset.

### Outcome measures

Primary outcome was functional outcome at 3 months after stroke on the mRS, ranging from 0 (no symptoms) to 6 (death).^14^ Secondary outcomes were functional independence, successful reperfusion and clinical improvement after intervention. Functional independence was defined as a 3-month mRS score of 0–2. Successful reperfusion was defined as an eTICI score of 2B (reperfusion of >50% of the previously occluded area) or higher. If completion angiography was not performed in two directions, reperfusion status was graded 2A at most. A decrease of ≥4 points on the National Institutes of Health Stroke Scale (NIHSS) between presentation and 24–48 hours postintervention or complete recovery (NIHSS 0) was considered a significant early clinical improvement.^15^ Safety outcomes were peri-interventional complications such as vessel dissections, perforations or other vascular injuries, vasospasms or new clots in different vascular territories, symptomatic intracranial haemorrhage and mortality at 3 months. Symptomatic intracranial haemorrhage was defined as haemorrhage related to neurological deterioration (decline of at least four points on the NIHSS) or death.^16^

### Missing data

Missing NIHSS scores were retrospectively scored with a standardised score chart that used the reported neurological examination information. The mRS score was assessed as part of usual care in all centres. Any follow-up mRS score of 0–5 assessed within 30 days was considered invalid and was replaced using multiple imputation. Multiple imputation was performed before selection of early and late window patients with the following variables: age, prestroke mRS, blood pressure, history of diabetes mellitus, myocardial infarction, previous stroke, atrial fibrillation or hypercholesterolaemia, NIHSS at presentation, ASPECTS at presentation, location of occlusion, collateral status, onset-to-groin time, reperfusion status, postintervention NIHSS and 3-month mRS.

### Statistical analysis

Baseline characteristics of late time window patients treated at or beyond 6.5 hours after symptom onset or time LSW were compared with those of early time window patients treated within 6.5 hours using χ^2^ tests for categorical variables and independent t-tests or Mann-Whitney U tests for continuous variables. Nearest-neighbour propensity score matching of late window and early window patients in a 1:2 ratio with a calliper width of 0.2 was performed with five imputed datasets to minimise the risk of confounding by indication.^17^ Late window and early window patients were matched on age, prestroke mRS, baseline NIHSS, baseline ASPECTS, location of occlusion, collateral status and treatment with intravenous thrombolysis (IVT) prior to EVT.^18^ Unmatched patients were excluded from further analysis. In each matched imputed dataset, logistic regression analyses were used to determine ORs for patients treated beyond compared with patients treated within the 6.5-hour time window. Effect estimates of regression analyses were corrected for the same variables as those used for matching to further minimise the risk of confounding. We estimated the pooled common OR as a measure of shift in the direction of a better outcome on the mRS. Pooled ORs were obtained using Rubin’s rules. Statistical analyses, multiple imputation and matching procedure were conducted with SPSS for Windows, V.24.0.

## Results

### Patient characteristics

**Figure 1:**
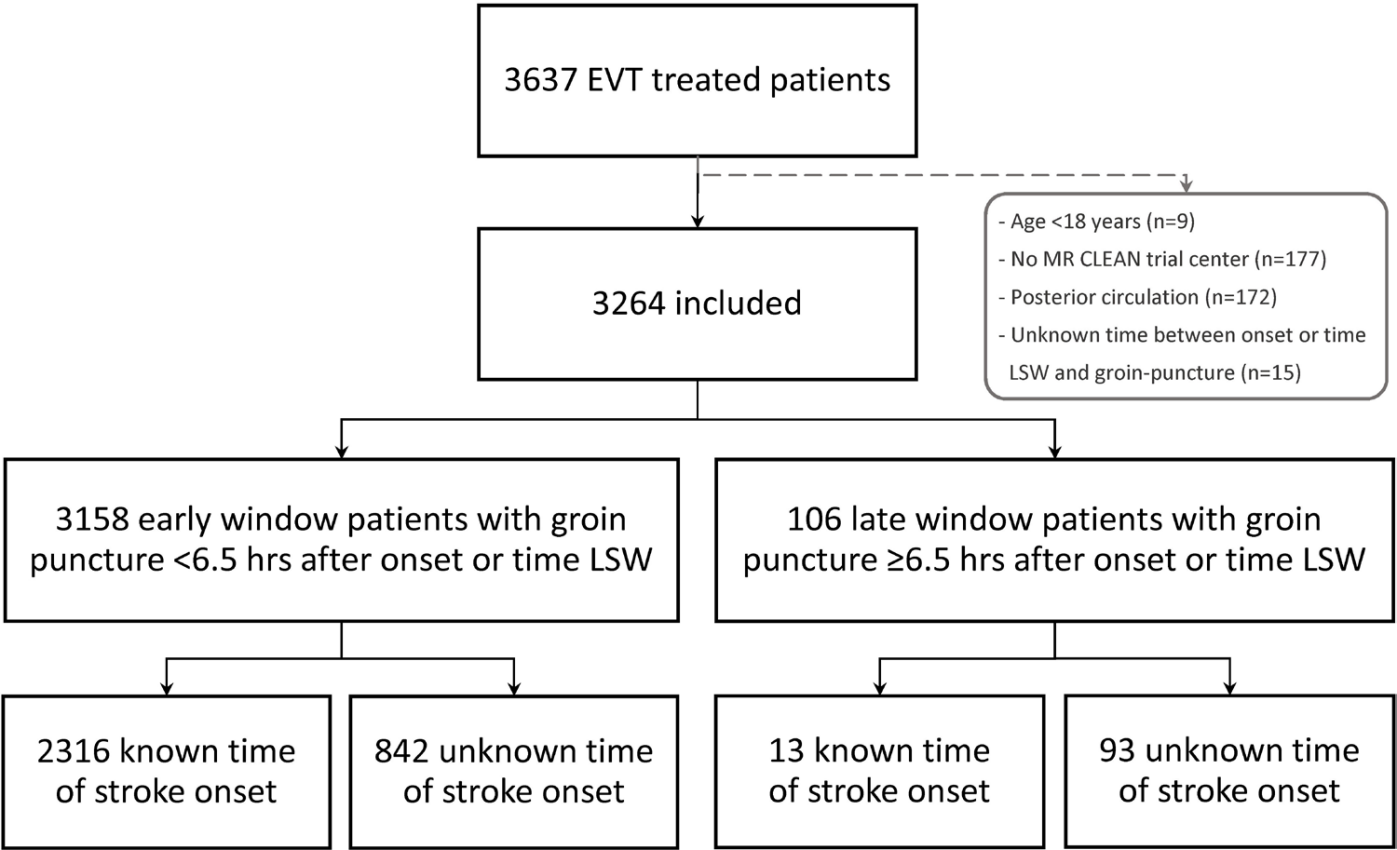
Flowchart of patients. EVT, endovascular treatment; LSW, last seen well; MR CLEAN, Multicenter Randomized Clinical Trial of Endovascular Treatment for Acute Ischemic Stroke in the Netherlands.

Of 3264 included patients, 3158 (96.8%) underwent EVT within 6.5 hours from symptom onset or LSW, of whom 842 (26.7%) had an unknown time of stroke onset. Of the 106 late window patients, the majority (n=93, 87.7%) had an unknown time of stroke onset (figure 1). Median time from onset or LSW to groin puncture was 195 min (IQR 150–250) for early window patients versus 508 min (IQR 415–637) for late window patients. Late window patients were slightly younger (67 vs 70 years, p=0.04) and received IVT less frequently (22.9% vs 76.8%, p<0.01) than early window patients. ASPECT-score at presentation was lower (median 8 vs 9, p<0.01) while collateral status was better for late window patients (dichotomised good collaterals filling >50%: 68.3% vs 57.7%, p=0.03). Of patients treated between 2016–2017, 15/65 late window patients (23.1%) underwent CT perfusion imaging prior to EVT versus 319/1673 (19.1%) in the early window (table 1).

**Table 1:** Baseline characteristics

	Early window patients with EVT <6.5 hours after onset or LSW (n=3158)	Late window patients with EVT ≥6.5 hours after onset or LSW (n=106)	P value*
Age (years); mean (SD)	70.0 (14.1)	67.1 (14.6)	**0.04**
Sex (male)	1642/3158 (52.0%)	47/106 (44.3%)	0.12
Prestroke mRS 0–1	2498/3087 (80.9%)	86/106 (81.1%)	0.96
Prestroke mRS 0–2	2729/3087 (88.4%)	96/106 (90.6%)	0.49
NIHSS at presentation; median (IQR)	16 (11–20) (n=3108)	16 (11–20) (n=103)	0.84
ASPECTS at presentation; median (IQR)	9 (7–10) (n=3055)	8 (6–10) (n=102)	**<0.01**
Location of occlusion			0.96
ICA	790/3004 (26.3%)	29/102 (28.4%)	
MCA M1-segment	1750/3004 (58.3%)	57/102 (55.9%)	
MCA M2-segment	440/3004 (14.6%)	15/102 (14.7%)	
Other (M3, ACA)	24/3004 (0.8%)	1/102 (1.0%)	
Collateral status			**0.01**
Absent collaterals	184/2959 (6.2%)	2/101 (2.0%)	
Filling ≤50% of occluded area	1067/2959 (36.1%)	30/101 (29.7%)	
Filling 51%–100% of occluded area	1146/2959 (38.7%)	38/101 (37.6%)	
Filling 100% of occluded area	562/2959 (19.0%)	31/101 (30.7%)	
CT perfusion performed	Treated 2014–2016: unknown	Treated 2014–2016: 6/41 (14.6%)	n/a
	Treated 2016–2017: 319/1673 (19.1%)	Treated 2016–2017: 15/65 (23.1%)	0.42
Transfer from primary hospital to intervention centre	1743/3158 (55.2%)	34/106 (32.1%)	**<0.01**
Treated with IVT	2420/3149 (76.8%)	24/105 (22.9%)	**<0.01**
Unknown time of onset	842/3158 (26.7%)	93/106 (87.7%)	**<0.01**
Time between onset/LSW and groin puncture in minutes; mean (SD); median (IQR)	203 (73) (n=3158) 195 (150–250)	559 (174) (n=106) 508 (415–637)	**<0.01**
Time between onset/LSW and end of procedure in minutes; mean (SD); median (IQR)	259 (78) (n=2925) 250 (200–312)	616 (185) (n=95) 564 (467–697)	**<0.01**
Performed procedure			0.07
Attempt for thrombectomy	2683/3149 (85.2%)	97/106 (91.5%)	
Catheterisation/DSA only	466/3149 (14.8%)	9/106 (8.5%)	

P-values <0.05 were considered significant and are provided in bold.

*χ^2^ tests for categorical variables and independent t-tests or Mann-Whitney U tests for continuous variables with complete data.

ACA, anterior cerebral artery; ASPECTS, Alberta Stroke Programme Early CT Score; DSA, digital subtraction angiography; EVT, endovascular treatment; ICA, internal carotid artery; IVT, intravenous thrombolysis; LSW, last seen well; MCA, middle cerebral artery; mRS, modified Rankin Scale; NIHSS, National Institutes of Health Stroke Scale.

**Figure 2:**
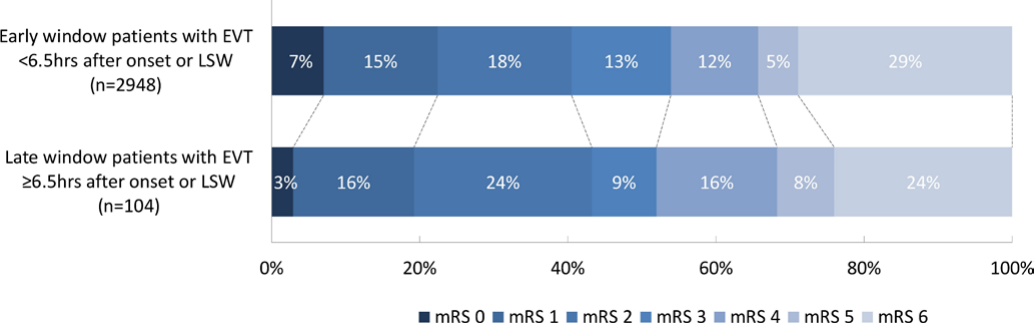
Distribution of mRS scores at 3 months. EVT, endovascular treatment; LSW, last seen well; mRS, modified Rankin Scale.

Of patients who underwent EVT outside the 6.5-hour time window, baseline characteristics were similar between those with known time of stroke onset and those with unknown time of stroke onset, except for a higher rate of IVT in known stroke onset patients (46.2% vs 19.6%, p=0.03) (online supplemental table 1). Patients treated beyond 6.5 hours who underwent CT perfusion imaging had lower NIHSS at presentation (median 13 vs 16, p=0.01) and appeared to have slightly lower ASPECTS at presentation (median 7 vs 9, p=0.09) compared with patients treated beyond 6.5 hours who did not undergo CT perfusion imaging, but all other characteristics were comparable (online supplemental table 2). Of all patients with a known time of stroke onset, there were no differences in baseline characteristics between those treated beyond versus within 6.5 hours, aside from a higher rate of IVT in the group treated in the early time window (79.0% vs 46.2%, p<0.01) (online supplemental table 3).

10.1136/svn-2020-000803.supp1 Supplementary data

### Clinical outcomes

Proportions of functional independence at 3 months (43.3% vs 40.5%, p=0.57), successful reperfusion (56.9% vs 61.7%, p=0.33) and mortality (24.0% vs 28.9%, p=0.28) were comparable between late and early window patients (table 2, figure 2).

**Table 2:** Outcome measures

	Early window patients with EVT <6.5 hours after onset or LSW (n=3158)	Late window patients with EVT ≥6.5 hours after onset or LSW (n=106)	P value*
Primary outcome			
Median 3-month mRS score (IQR)	3 (2–6) (n=2948)	3 (2–5) (n=104)	0.92
Secondary outcomes			
Functional independence (3-month mRS 0–2)	40.5% (1193/2948)	43.3% (45/104)	0.57
Successful reperfusion (eTICI ≥2B)	61.7% (1897/3076)	56.9% (58/102)	0.33
Significant early clinical improvement (≥4 points decrease on NIHSS or NIHSS 0)	54.7% (1542/2820)	46.5% (46/99)	0.11
Safety outcomes			
Peri-interventional complications	8.7% (274/3158)	9.4% (10/106)	0.79
Symptomatic intracranial haemorrhage	5.9% (185/3158)	4.7% (5/106)	0.62
Mortality at 3 months	28.9% (853/2948)	24.0% (25/104)	0.28

*Mann-Whitney U test for difference in 3-month mRS score and χ^2^ tests for difference in proportions of other outcomes.

eTICI, extended Treatment In Cerebral Infarction; EVT, endovascular treatment; LSW, last seen well; mRS, modified Rankin Scale; NIHSS, National Institutes of Health Stroke Scale.

Logistic regression analysis showed no difference in outcomes (online supplemental table 4). Overall, 4.0% of data points in the imputation model were missing, and the proportion of missing mRS scores was 6.5%. After nearest-neighbour propensity score matching of five imputed datasets in a 1:2 ratio, 96.4% of the late window patients could be matched to two early window patients. Baseline characteristics of the five matched datasets are described in online supplemental table 5. Adjusted logistic regression after matching showed no significant difference in distribution of the mRS between the late window patients and their matched early window patients (pooled adjusted common OR for a shift of 1 point improvement in mRS 1.04, 95% CI 0.56 to 1.93) (table 3). Furthermore, proportions of functional independence at 3 months (pooled adjusted OR (aOR) 1.17, 95% CI 0.58 to 2.37), successful reperfusion (aOR 0.75, 95% CI 0.41 to 1.39) and postintervention significant early clinical improvement (aOR 0.79, 95% CI 0.44 to 1.43) were similar. Concerning safety parameters, no significant difference was found in rates of peri-interventional complications (aOR 1.52, 95% CI 0.50 to 4.58), symptomatic intracranial haemorrhage (aOR 1.03, 95% CI 0.26 to 4.04) or mortality (aOR 0.76, 95% CI 0.32 to 1.79) (table 3).

**Table 3:** Logistic regression analysis in matched patients

	Matched late window patients with EVT ≥6.5 hours vs early window patients with EVT <6.5 hours after onset or LSW*			
	Unadjusted OR (95% CI)	P value	Adjusted OR (95% CI)†	P value
Primary outcome				
3-month mRS score reduction (shift analysis)‡	0.99 (0.59 to 1.67)	0.98	1.04 (0.56 to 1.93)	0.89
Secondary outcomes				
Functional independence (3-month mRS 0–2)	1.05 (0.63 to 1.75)	0.87	1.17 (0.58 to 2.37)	0.65
Successful reperfusion (eTICI ≥2B)	0.75 (0.41 to 1.38)	0.35	0.75 (0.41 to 1.39)	0.35
Significant early clinical improvement (≥4 points decrease on NIHSS or NIHSS 0)	0.78 (0.45 to 1.32)	0.35	0.79 (0.44 to 1.43)	0.43
Safety outcomes				
Peri-interventional complications	1.49 (0.52 to 4.24)	0.45	1.52 (0.50 to 4.58)	0.45
Symptomatic intracranial haemorrhage	0.99 (0.28 to 3.52)	0.98	1.03 (0.26 to 4.04)	0.97
Mortality at 3 months	0.84 (0.43 to 1.65)	0.61	0.76 (0.32 to 1.79)	0.52

*Matched on: age, prestroke mRS, NIHSS at presentation, ASPECTS at presentation, location of occlusion, collateral status and treatment with IVT. With a calliper of 0.2, 511 (96.4%) of the 530 late window patients treated beyond 6.5 hours (5 datasets with 106 late window patients per dataset) could be matched in a 1:2 ratio to 1022 early window patients treated within 6.5 hours. ORs were pooled from the five matched datasets using Rubin’s rules.

†Adjusted for: age, prestroke mRS, NIHSS at presentation, ASPECTS at presentation, location of occlusion, collateral status and treatment with IVT.

‡Common OR indicating the odds of improvement of 1 point on the mRS.

eTICI, extended Treatment In Cerebral Infarction; EVT, endovascular treatment; LSW, last seen well; mRS, modified Rankin Scale; NIHSS, National Institutes of Health Stroke Scale.

Of patients treated beyond 6.5 hours, there were no differences in outcomes between those with known and unknown time of stroke onset (online supplemental table 1). Those who underwent CT perfusion imaging more often had symptomatic intracranial haemorrhage (14.3% vs 2.4%, p=0.02), but all other outcomes were similar (online supplemental table 2). Finally, no differences were found between outcomes of patients with known time of stroke onset treated either beyond versus within 6.5 hours (online supplemental table 3).

## Discussion

We observed similar rates of functional independence, successful reperfusion and safety outcomes in selected patients with favourable characteristics, such as good collaterals, treated with EVT beyond 6.5 hours from symptom onset or time LSW compared with patients treated within 6.5 hours without mandatory perfusion imaging.

Although several studies showed that EVT is safe and effective in late window patients selected using only non-contrast CT and CT angiography,^19 20^ the role of ASPECTS and collateral status compared with MR diffusion or CT perfusion imaging requires further study. Previous studies showed no additional value of CT perfusion imaging in either prognostication of 3-month mRS after EVT compared with non-contrast CT ASPECT score,^21^ or in selection of patients with good outcomes after EVT beyond 6 hours compared with selection using solely non-contrast CT and CT angiography.^22–25^ Two other studies showed that a substantial proportion of DAWN-ineligible and DEFUSE 3-ineligible patients also reached functional independence at 3 months after EVT.^26 27^ Our findings confirm that in clinical practice similar outcomes can be obtained in selected patients without additional determination of core and penumbra. Randomised trials without mandatory perfusion imaging, such as the ongoing pragmatic MR CLEAN-LATE,^28^ which is based on collateral and ASPECT scoring, may be able to identify more patients who can still benefit from EVT outside the 6-hour time window and deliver further modifications in guidelines for patients who had a stroke in the late time window.^29^

A previous analysis of the MR CLEAN Registry showed that in patients treated within 6.5 hours, every hour of delay resulted in a 5.3% decrease of probability of functional independence.^6^ However, that analysis excluded late window patients treated beyond 6.5 hours after onset and only patients treated before June 2016 were included. In the present study, only 3.2% of patients were treated beyond 6.5 hours and we observed similar outcomes compared with patients treated within 6.5 hours, which suggests patient selection. Although the specifics of CT perfusion scans of patients have not yet been assessed, it is unlikely that these had much influence on patient selection or outcome since we found similar low rates of the use of CT perfusion in early and late window patients. Furthermore, our patients were treated before the publication of the DAWN/DEFUSE 3 trials, in a time when perfusion imaging was not common practice and patients presenting beyond 6 hours of onset or time LSW were generally excluded from EVT, which may also explain the low proportion of late window patients. We found that late window patients were younger and had better collateral status. This suggests selection on increased collateral supply, which a post hoc analysis of the original MR CLEAN study showed to be strongly associated with better outcome after EVT,^30^ and may imply that those patients have more salvageable tissue.

The majority of late window patients in this study had an unknown time of stroke onset. This is similar to the population treated in the DAWN and DEFUSE 3 studies (respectively, 90% and 66% unknown onset). There are indications that in wake-up strokes, stroke onset occurs shortly before awakening.^31^ This implies that the actual time between onset and EVT may well be within the accepted time window. Therefore, it would be interesting to see if similar results can be obtained in known stroke onset patients treated >6 hours after onset and wake-up patients who had a stroke treated >6 hours after awakening. Although we found no differences between characteristics and outcomes of patients with known versus unknown time of stroke onset treated beyond 6.5 hours, nor of known-onset patients who had a stroke treated either beyond versus within 6.5 hours, our study is limited by the fact that only 12.3% of patients had documented known-onset stroke in the late window.

The main strength of our observational study is that it was performed with data from a large, nationwide EVT registry from routine clinical practice without prespecified imaging selection other than an anterior circulation occlusion. Furthermore, we used adjusted logistic regression after propensity score matching to minimalise the risk of differences in outcome between patients treated within versus beyond the 6.5-hour time window due to differences in baseline characteristics. An important limitation of this study is that no records were kept of the group of untreated late window patients. Therefore, the exact level and method of patient selection cannot be assessed and, although outcomes are similar to those of early window patients, no evidence of clinical benefit can be established without a control group. Furthermore, since there is currently no data available concerning the specific CT perfusion results, it is not possible to compare penumbra and core status between early and late window patients. However, it is unlikely that this influenced overall outcomes, since only a small minority of patients underwent CT perfusion imaging.

## Conclusions

Without the use of the DAWN or DEFUSE 3 trial CT perfusion selection criteria, EVT in the 6.5–24-hour time window was safe and not associated with poorer outcome in selected patients with favourable clinical and CT/CTA characteristics. Randomised controlled trials with more lenient inclusion criteria than the DAWN and DEFUSE 3 are needed to identify more patients who can benefit from EVT in the late window.

## Data Availability

Individual patient data cannot be made available, because no patient approval has been obtained for sharing data, even in coded form. However, syntax and output files of statistical analyses can be made available upon reasonable request.
