# Supplementary material for: Endovascular treatment in anterior circulation stroke beyond 6.5 hours after onset or time last seen well: results from the MR CLEAN Registry
Source: Stroke Vasc Neurol. 2021 Apr 7;6(4):572–80. doi: 10.1136/svn-2020-000803 (PMC8717786; doi:10.1136/svn-2020-000803)
Supplement: Supplementary data [file svn-2020-000803supp001.pdf]

SUPPLEMENTAL MATERIAL

Supplemental table 1. Baseline characteristics and outcomes of late window patients with EVT ≥6.5 hours after known time of stroke onset versus late window patients with EVT ≥6.5 hours after unknown time of stroke onset.

	Late window patients with EVT ≥6.5 hours after known time of stroke onset (n=13)	Late window patients with EVT ≥6.5 hours after unknown time of stroke onset (LSW) (n=93)	*p* value*
**Baseline characteristics**			
Age (years); mean (SD)	67.7 (17.4)	67.0 (14.3)	0.87
Sex (male)	3/13 (23.1%)	44/93 (47.3%)	0.10
Pre-stroke mRS 0-1	11/13 (84.6%)	75/93 (80.6%)	0.73
Pre-stroke mRS 0-2	0	83/93 (89.2%)	0.21
NIHSS at presentation; median [IQR]	15 [9-19] (n=12)	16 [11-20] (n=91)	0.47
ASPECTS at presentation; median [IQR]	9 [7-10] (n=13)	8 [6-10] (n=89)	0.24
Location of occlusion			0.68
ICA	2/13 (15.4%)	27/89 (30.3%)	
MCA M1-segment	9/13 (69.2%)	48/89 (53.9%)	
MCA M2-segment	2/13 (15.4%)	13/89 (14.6%)	
Other (M3, ACA)	0	1/89 (1.1%)	
Collateral status			0.61
Absent collaterals	0	2/88 (2.3%)	
Filling ≤50% of occluded area	3/13 (23.1%)	27/88 (30.7%)	
Filling 51-100% of occluded area	7/13 (53.8%)	31/88 (35.2%)	
Filling 100% of occluded area	3/13 (23.1%)	28/88 (31.8%)	
CT perfusion performed	2/13 (15.4%)	19/93 (20.4%)	0.67
Transfer from primary hospital to intervention center	9/13 (69.2%)	25/93 (26.9%)	**<0.01**
Treated with IVT	6/13 (46.2%)	18/92 (19.6%)	**0.03**

Dekker L, *et al. Stroke Vasc Neurol* 2021; 6:e000803. doi: 10.1136/svn-2020-000803

Time between onset/LSW and groin puncture in minutes; mean (SD); median [IQR]	516 (172) (n=13) 449 [405-553]	565 (175) (n=93) 518 [420-654]	0.34
Time between onset/LSW and sheath from groin in minutes; mean (SD); median [IQR]	501 (63) (n=9) 481 [455-548]	628 (189) (n=86) 578 [469-733]	**0.05**
Performed procedure			0.91
Attempt for thrombectomy	12/13 (92.3%)	85/93 (91.4%)	
Catheterization/DSA only	1/13 (7.7%)	8/93 (8.6%)	
**Outcomes**			*p* value†
Primary outcome			
Median 3-month mRS score [IQR]	2 [1-5] (n=13)	3 [2-5] (n=91)	0.52
Secondary outcomes			
Functional independence (3-month mRS 0-2)	61.5% (8/13)	40.7% (37/91)	0.16
Successful reperfusion (eTICI ≥2B)	46.2% (6/13)	58.4% (52/89)	0.40
Significant early clinical improvement (≥4 points decrease on NIHSS or NIHSS 0)	45.5% (5/11)	46.6% (41/88)	0.94
Safety outcomes			
Peri-interventional complications	7.7% (1/13)	9.7% (9/93)	0.82
Symptomatic intracranial hemorrhage	7.7% (1/13)	4.3% (4/93)	0.59
Mortality at 3 months	23.1% (3/13)	24.2% (22/91)	0.93

*Chi-square tests for categorical variables and independent t-tests or Mann-Whitney U tests for continuous variables with complete data

Dekker L, *et al. Stroke Vasc Neurol* 2021; 6:e000803. doi: 10.1136/svn-2020-000803

†Mann-Whitney U test for difference in 3-month mRS score and chi-square tests for difference in proportions of other outcomes

EVT: endovascular treatment; SD: standard deviation; mRS: modified Rankin Scale; NIHSS: National Institutes of Health Stroke Scale; IQR: interquartile range; ASPECTS: Alberta Stroke Programme Early CT Score; ICA: internal carotid artery; MCA: middle cerebral artery; ACA: anterior cerebral artery; IVT: intravenous thrombolysis; LSW: last seen well; DSA: digital subtraction angiography; eTICI: extended Treatment In Cerebral Infarction

Supplemental table 2. Baseline characteristics and outcomes of late window patients with EVT ≥6.5hrs who received CT perfusion imaging vs. late window patients with EVT ≥6.5hrs who did not receive CT perfusion imaging

	Late window patients with EVT ≥6.5hrs with CT perfusion imaging (n=21)	Late window patients with EVT ≥6.5hrs without CT perfusion imaging (n=85)	*p* value*
**Baseline characteristics**			
Age (years); mean (SD)	65.8 (14.4)	67.4 (14.7)	0.65
Sex (male)	10/21 (47.6%)	37/85 (43.5%)	0.74
Pre-stroke mRS 0-1	18/21 (85.7%)	68/85 (80.0%)	0.55
Pre-stroke mRS 0-2	20/21 (95.2%)	76/85 (89.4%)	0.41
NIHSS at presentation; median [IQR]	13 [8-17] (n=19)	16 [12-20] (n=84)	**0.01**
ASPECTS at presentation; median [IQR]	7 [5-9] (n=20)	9 [7-10] (n=82)	*0.09*
Location of occlusion			0.12
ICA	5/21 (23.8%)	24/81 (29.6%)	
MCA M1-segment	10/21 (47.6%)	47/81 (58.0%)	
MCA M2-segment	5/21 (23.8%)	10/81 (12.3%)	
Other (M3, ACA)	1/21 (4.8%)	0	
Collateral status			0.80
Absent collaterals	0	2/82 (2.4%)	
Filling ≤50% of occluded area	6/19 (31.6%)	24/82 (29.3%)	
Filling 51-100% of occluded area	6/19 (31.6%)	32/82 (39.0%)	
Filling 100% of occluded area	7/19 (36.8%)	24/82 (29.3%)	
Treated with IVT	4/21 (19.0%)	20/84 (23.8%)	0.64
Unknown time of onset	19/21 (90.5%)	74/85 (87.1%)	0.67
Time between onset/LSW and groin puncture in minutes; mean (SD); median [IQR]	540 (146) (n=21) 518 [420-609]	563 (181) (n=85) 505 [415-664]	0.58
Time between onset/LSW and sheath from groin in minutes; mean (SD); median [IQR]	577 (159) (n=18) 523 [478-649]	625 (190) (n=77) 571 [463-719]	0.33
Performed procedure			0.85
	19/21 (90.5%)	78/85 (91.8%)	

Dekker L, *et al. Stroke Vasc Neurol* 2021; 6:e000803. doi: 10.1136/svn-2020-000803

Attempt for thrombectomy	2/21 (9.5%)	7/85 (8.2%)	
Catheterization/DSA only			
**Outcomes**			*p* value†
Primary outcome			
Median 3-month mRS score [IQR]	2 [2-4] (n=21)	3 [2-6] (n=83)	0.83
Secondary outcomes			
Functional independence (3-month mRS 0-2)	42.9% (9/21)	43.4% (36/83)	0.97
Successful reperfusion (eTICI ≥2B)	68.4% (13/19)	54.2% (45/83)	0.26
Significant early clinical improvement (≥4 points decrease on NIHSS or NIHSS 0)	27.8% (5/18)	50.6% (41/81)	*0.08*
Safety outcomes			
Peri-interventional complications	9.5% (2/21)	9.4% (8/85)	0.99
Symptomatic intracranial hemorrhage	14.3% (3/21)	2.4% (2/85)	**0.02**
Mortality at 3 months	14.3% (3/21)	26.5% (22/83)	0.24

*Chi-square tests for categorical variables and independent t-tests or Mann-Whitney U tests for continuous variables with complete data.

†Mann-Whitney U test for difference in 3-month mRS score and chi-square tests for difference in proportions of other outcomes

EVT: endovascular treatment; SD: standard deviation; mRS: modified Rankin Scale; NIHSS: National Institutes of Health Stroke Scale; IQR: interquartile range; ASPECTS: Alberta Stroke Program Early CT Score; ICA: internal carotid artery; MCA: middle cerebral artery; ACA: anterior cerebral artery; IVT: intravenous thrombolysis; LSW: last seen well; DSA: digital subtraction angiography; eTICI: extended Treatment In Cerebral Ischemia

Supplemental table 3. Baseline characteristics and outcomes of patients with known time of stroke onset treated with EVT beyond versus within 6.5 hours.

	Late window patients with EVT ≥6.5 hours after known time of stroke onset (n=13)	Early window patients with EVT <6.5 hours after known time of stroke onset (n=2316)	*p* value*
**Baseline characteristics**			
Age (years); mean (SD)	67.7 (17.4)	69.3 (14.1)	0.69
Sex (male)	3/13 (23.1%)	1246/2316 (53.8%)	**0.03**
Pre-stroke mRS 0-1	11/13 (84.6%)	1879/2269 (82.8%)	0.86
Pre-stroke mRS 0-2	0	2038/2269 (89.8%)	0.23
NIHSS at presentation; median [IQR]	15 [9-19] (n=12)	15 [11-19] (n=2278)	0.72
ASPECTS at presentation; median [IQR]	9 [7-10] (n=13)	9 [8-10] (n=2245)	0.90
Location of occlusion			0.80
ICA	2/13 (15.4%)	589/2203 (26.7%)	
MCA M1-segment	9/13 (69.2%)	1276/2203 (57.9%)	
MCA M2-segment	2/13 (15.4%)	322/2203 (14.6%)	
Other (M3, ACA)	0	16/2203 (0.7%)	
Collateral status			0.55
Absent collaterals	0	127/2169 (5.9%)	
Filling ≤50% of occluded area	3/13 (23.1%)	768/2169 (35.4%)	
Filling 51-100% of occluded area	7/13 (53.8%)	853/2169 (39.3%)	
Filling 100% of occluded area	3/13 (23.1%)	421/2169 (19.4%)	
CT perfusion performed	2/13 (15.4%)	218/1201 (18.2%)	0.80
Transfer from another hospital to intervention center	9/13 (69.2%)	1306/2316 (56.4%)	0.35
Treated with IVT	6/13 (46.2%)	1825/2309 (79.0%)	**<0.01**
Time between onset/LSW and groin puncture in minutes; mean (SD); median [IQR]	516 (172) (n=13)<br>449 [405-553]	187 (64) (n=2316)<br>180 [140-225]	**<0.01**

Time between onset/LSW and sheath from groin in minutes; mean (SD); median [IQR]	501 (63) (n=9) 481 [455-548]	243 (72) (n=2147) 235 [190-290]	**<0.01**
Performed procedure			0.48
Attempt for thrombectomy	12/13 (92.3%)	1972/2308 (85.4%)	
Catheterization/DSA only	1/13 (7.7%)	336/2308 (14.6%)	
**Outcomes**			*p* value†
Primary outcome			
Median 3-month mRS score [IQR]	2 [1.5-5.5] (n=13)	3 [2-6] (n=2155)	0.65
Secondary outcomes			
Functional independence (3-month mRS 0-2)	61.5% (8/13)	43.3% (933/2155)	0.19
Successful reperfusion (eTICI ≥2B)	46.2% (6/13)	61.2% (1377/2251)	0.27
Significant early clinical improvement (≥4 points decrease on NIHSS or NIHSS 0)	45.5% (5/11)	54.9% (1136/2068)	0.53
Safety outcomes			
Peri-interventional complications	7.7% (1/13)	9.0% (208/2316)	0.87
Symptomatic intracranial hemorrhage	7.7% (1/13)	6.0% (139/2316)	0.80
Mortality at 3 months	23.1% (3/13)	27.0% (581/2155)	0.75

*Chi-square tests for categorical variables and independent t-tests or Mann-Whitney U tests for continuous variables with complete data

†Mann-Whitney U test for difference in 3-month mRS score and chi-square tests for difference in proportions of other outcomes

EVT: endovascular treatment; SD: standard deviation; mRS: modified Rankin Scale; NIHSS: National Institutes of Health Stroke Scale; IQR: interquartile range; ASPECTS: Alberta Stroke Programme Early CT Score; ICA: internal carotid artery; MCA: middle cerebral artery; ACA: anterior cerebral artery; IVT: intravenous thrombolysis; LSW: last seen well; DSA: digital subtraction angiography; eTICI: extended Treatment In Cerebral Infarction

Supplemental table 4. Logistic regression analysis for late vs. early window patients.

	Late window patients with EVT ≥6.5 hours (n=106) versus early window patients with EVT <6.5 hours (n=3158) after onset or LSW			
	unadjusted OR (95% CI)	*p* value	adjusted OR (95% CI)*	*p* value
Primary outcome				
3-month mRS score reduction (shift analysis) †	1.02 (0.72-1.44)	0.92	0.86 (0.58-1.28)	0.46
Secondary outcomes				
Functional independence (3-month mRS 0-2)	1.12 (0.76-1.67)	0.57	0.89 (0.53-1.49)	0.65
Successful reperfusion (eTICI ≥2B)	0.83 (0.56-1.24)	0.36	0.85 (0.54-1.32)	0.46
Significant early clinical improvement (≥4 points decrease on NIHSS or NIHSS 0)	0.72 (0.48-1.07)	0.11	0.72 (0.46-1.14)	0.17
Safety outcomes				
Peri-interventional complications	1.10 (0.57-2.13)	0.79	1.06 (0.49-2.27)	0.89
Symptomatic intracranial hemorrhage	0.80 (0.32-1.98)	0.62	0.84 (0.30-2.39)	0.75
Mortality at 3 months	0.78 (0.49-1.23)	0.28	0.88 (0.50-1.57)	0.67

*Adjusted for: age, pre-stroke mRS, NIHSS at presentation, ASPECTS at presentation, location of occlusion, collateral status and treatment with IVT

†Common odds ratio indicating the odds of improvement of 1 point on the mRS

Dekker L, *et al. Stroke Vasc Neurol* 2021; 6:e000803. doi: 10.1136/svn-2020-000803

EVT: endovascular treatment; LSW: last seen well; OR: odds ratio; CI: confidence interval; mRS: modified Rankin Scale; eTICI: extended Treatment In Cerebral Infarction; NIHSS: National Institutes of Health Stroke Scale

Supplemental table 5. Baseline characteristics of matched early window vs. late window patients of the 5 imputed datasets.

	Matched early window patients with EVT <6.5 hours after onset or LSW (n=1022)*	Matched late window patients with EVT ≥6.5 hours after onset or LSW (n=511)*
Age (years); mean (SD)	67.4 (14.5)	67.7 (14.4)
Sex (male)	546 (53.4%)	224 (43.8%)
Pre-stroke mRS 0-1	836 (81.8%)	411 (80.4%)
Pre-stroke mRS 0-2	932 (91.2%)	461 (90.2%)
NIHSS at presentation; median [IQR]	16 [11-20]	15 [11-20]
ASPECTS at presentation; median [IQR]	8 [7-10]	8 [6-9]
Location of occlusion		
ICA	282 (27.6%)	138 (27.0%)
MCA M1-segment	582 (56.9%)	291 (56.9%)
MCA M2-segment	143 (14.0%)	76 (14.9%)
Other (M3, ACA)	15 (1.5%)	6 (1.2%)
Collateral status		
Absent collaterals	23 (2.3%)	11 (2.2%)
Filling ≤50% of occluded area	284 (27.8%)	153 (29.9%)
Filling 51-100% of occluded area	384 (37.6%)	193 (37.8%)
Filling 100% of occluded area	331 (32.4%)	154 (30.1%)
Treated with IVT	266 (26.0%)	124 (24.3%)

Dekker L, *et al. Stroke Vasc Neurol* 2021; 6:e000803. doi: 10.1136/svn-2020-000803

Time between onset/LSW and groin puncture in minutes; mean (SD); median [IQR]	210 (81)<br>195 [150-270]	557 (175)<br>505 [415-634]
Time between onset/LSW and sheath from groin in minutes; mean (SD); median [IQR]	267 (89)<br>255 [196-331]	592 (195)<br>548 [459-672]

*With a caliper of 0.2, 511 (96.4%) of the 530 late window patients with EVT ≥6.5 hours (5 datasets with 106 late window patients per dataset) could be matched in a 1:2 ratio to 1022 early window patients with EVT <6.5 hours after onset or LSW.

EVT: endovascular treatment; LSW: last seen well; SD: standard deviation; mRS: modified Rankin Scale; NIHSS: National Institutes of Health Stroke Scale; IQR: interquartile range; ASPECTS: Alberta Stroke Programme Early CT Score; IQR: interquartile range; ICA: internal carotid artery; MCA: middle cerebral artery; ACA: anterior cerebral artery; IVT: intravenous thrombolysis
